# Mobile Dental Radiology—Evaluation of Quality Requirements for Radiographs Applying Handheld Mobile Radiography

**DOI:** 10.1111/ger.70023

**Published:** 2025-09-26

**Authors:** Amina Maria Geibel, Constanze Keutel, Daniela Kildal, Margrit‐Ann Geibel

**Affiliations:** ^1^ Department of Oral Surgery University Center for Dental Medicine, University of Basel Basel Switzerland; ^2^ Department of Oral and Maxillofacial Surgery, Section of Dento‐Maxillofacial Radiology University Hospital Tübingen Germany; ^3^ Department of Diagnostic and Interventional Radiology University Hospital Ulm Ulm Germany; ^4^ Radiology, Upper Valais Hospital Center (SZO), Hôpital du Valais Brig Switzerland; ^5^ Department of Oral and Maxillofacial Surgery, Dento‐ and Maxillofacial Radiology University Ulm Germany; ^6^ Department Gender‐Specific Dentistry Danube University Krems Austria

**Keywords:** dental radiology, handheld radiography, intraoral radiology, mobile dentistry

## Abstract

**Background:**

Radiography is required in domiciliary dentistry in order to ensure accurate diagnosis, but the available devices have not yet been approved due to a lack of research regarding the quality of single tooth radiography.

**Aim:**

To examine dental radiographs taken using the mobile X‐ray device (Nomad Pro 2, Envista, Brea, CA) with regard to their quality, examinability and their benefit for treatment planning in mobile dentistry.

**Methods:**

The image quality of radiographs using a mobile handheld X‐ray system was assessed. The quality criteria catalogue of the German Dental Association was applied.

**Results:**

Of 127 dental radiographs, 80% (101) showed no quality defects. Diagnosis‐relevant quality defects were found in 17% (21). 61% (78) of radiographs had clinically relevant secondary findings that had an influence on further dental treatment and on the prognosis of the teeth.

**Conclusions:**

Dental care in nursing homes presents significant challenges, particularly regarding oral preventive medicine. Surgical procedures are legally contestable without dental radiology, and this raises professional and ethical concerns. Mobile dental radiology is an important component of treatment in mobile dentistry. Unexpected findings such as apical osteolysis or root remnants can only be identified and treated by means of radiography.

## 1 Introduction

Worldwide, 30% of people in need of care require support for their oral health, 60% of people in need of care are no longer able to visit a dental practice [1]. Demographic developments mean that a further increase in the number of people requiring treatment at home or in retirement and nursing homes can be expected in the coming years [1]. The oral health care of these patients could be improved using domiciliary dental care, where dental treatment is carried out on site (retirement home, rehabilitation centre) [2, 3]. Radiography is also required in mobile dentistry in order to ensure accurate diagnosis.

In Germany, radiography is covered by dental care in case clinical examination cannot provide a sufficient or appropriate diagnosis [4, 5]. It can be difficult or even impossible to apply dental radiography to immobile, seriously ill or disabled patients. However, with a mobile dental X‐ray unit, comprehensive dental diagnostics can be achieved in difficult environments such as retirement or nursing homes with several beds.

Currently available X‐ray systems that are developed for intraoral applications are so‐called handheld X‐ray devices. In the United States, Netherlands and Sweden, in particular, the Nomad Pro2 from Dexis/KaVo is widely used. A mobile X‐ray device enables the dentist to assess the need for surgical or more complex dental measures and to provide precise treatment plans more quickly for non‐mobile patients. As such, the conditions in mobile dentistry should match the standards of quality in ambulatory dental practice.

With respect to radiation exposure, a statistically significant increase in cancer rates has not been detected in populations exposed to doses of less than 0.05 Sv [6]. The dose‐related aspects in mobile radiology using handheld devices have been investigated internationally, with the conclusion, that the effective dose in mobile radiology is comparable to that from wall‐mounted devices or that it is even possible to use a lower dose. Likewise, radiation exposure from mobile radiography has been measured for the investigator. Exposure remained under the specified maximum dose for all investigators who used the handheld device [7, 8, 9, 10]. ‘When CE‐certified portable devices are used with rectangular collimation and a backshatter radiation shield with adapted technique resulting in a beam parallel to the ground, operator exposure stays well within dose limits’ according to Hoogeveen & Berkhout (2022) [11].

The authorisation requirements for mobile X‐ray devices differ among significantly between countries. The available devices are not yet approved in Germany due, among other things, to the lack of studies on quality. Accordingly, the objective of our study was to evaluate the image quality and determine the dental benefits that can be achieved with a portable mobile dental X‐ray machine for non‐mobile patients in routine clinical practice.

## 2 Methods

A total of 127 dental single tooth radiographs taken using the Nomad Pro 2 were evaluated. All images were taken by an experienced dentist who is familiar with the Nomad Pro 2 in a retirement home or nursing home setting for the purpose of dental diagnosis in the course of routine dental care.

Written informed consent was obtained from participants (or their legal guardian/next of kin); informed consent and patient anonymity are preserved. The justifying indication and the clinical findings were collected by the treating dentist. The images were anonymised. A key file was created containing patient names and image numbers, which is only accessible by the treating dentist. The images were accessed by three dentists with different specialisations and professional experience: one specialist in oral and maxillofacial surgery, one specialist in oral surgery and one in the second year of training in conservative dentistry. The influence of their professional experience on the findings was investigated.

The following questions had to be answered for the assessment of the dental radiographs taken with a mobile hand‐held X‐ray unit: Is the radiograph image relevant for the treatment, Are the defined quality criteria fulfilled, Is the field of view compatible with the justifying indication and Were other findings such as secondary or incidental findings detected?

The results were compared with data from published studies that had used a conventional wall‐mounted dental radiography device. The assessment took place under standard ambient lighting on a monitor approved for examining radiographs. All three dentists assessed the images according to the standard approach of the German Federal Dental Association [12] according to quality criteria (see Table 1) such as examinability and quality defects.

**TABLE 1:** Check list and criteria for assessing the image quality.

Variable		Quality criteria	Relevant for treatment
Approximal surface of crown region	Yes/no	x	x
Alveolar border	Yes/no	x	x
Dentine‐enamel junction	Yes/no	x	x
Dental pulp chamber and root canals	Yes/no	x	x
Interdental/interradicular septum	Yes/no	x	x
Periodontal ligament	Yes/no	x	x
Bone structure of the periapical/periradicular area	Yes/no	x	x
Is the image detail suitable for diagnosis?	Yes/no	x	
Is the image section relevant to justifying indication?	Yes/no	x	
Quality defects relevant to diagnosis	Yes/no	x	
Secondary findings related to justifying indication	Yes/no	x	

Indications justifying dental imaging in a patient were root remnants (surgery), caries examination, periodontitis examination and implantology.

First, special focus was set on relevance for treatment in the assessment of the osseous structures, such as the alveolar border, the interradicular septum, the assessability of the periodontal ligament (PDL) and the periapical bone structure.

The criteria catalogue of the German Dental Association was used to assess image quality: Is the central beam correctly centred, Is the phosphor storage plate (PSP)/image plate free of quality defects, Are there any foreign bodies in the beam path or are other overlaps visible and are contrast and sharpness sufficient? This allowed the identified quality defects to be precisely differentiated.

The defects in the radiograph were recorded and evaluated individually (Table 3). Cracks, creases, defects and signs of ageing were regarded as quality defects on the phosphor storage plates.

Examples of the classification used in this study (see also Table 1 for our checklist and criteria for assessing the image quality) were: Tartar was classified as a finding and not as an incidental or secondary finding; for ankylosed teeth were classified as a radiological finding; if the justifying indication included both the initial examination and caries in the study question, caries was recorded and the cementoenamel junction was considered recognisable even in the presence of root remnants if the cementoenamel junction was sufficiently visible with a crown present.

The Nomad Pro 2 used in our study operates at 60 kV and 2.5 mA. According to the specifications of the German Dental Association, imaging voltages of ≥ 60 kV, a resolution of ≥ 5 line pairs/mm, and a distance between the focus and the tube end of ≥ 200 mm are standard for dental X‐ray diagnostics [13]. The average effective dose is approximately 0.005 mSv for analogue dental radiographs and 0.003 mSv for digital dental radiographs [14].

The data from this study using a mobile handheld X‐ray device on patients in retirement or nursing homes were compared with those from a published study from 2012 in which various wall‐mounted X‐ray devices were examined at a university hospital [15].

The statistical method was descriptive, and the results were presented in charts.

## 3 Results

A total of 127 dental radiographs were examined. Overall, their quality was very good; 101 (80%) radiographs showed no qualitative limitations. The bone structure of the periradicular/periapical area was found to be diagnostic in 109 (86%) radiographs. In fewer than 4% of the radiographs, the bony structures such as the alveolar ridge and the tooth structure (dentin–enamel junction, dental pulp chamber, etc.) could not be assessed with sufficient accuracy (Table 2).

**TABLE 2:** Recorded image quality defects.

Check list	% of found defects
Proximal coronal area	4%
Alveolar border	2%
Dentinoenamel junction	2%
Pulp cavity and root canals	2%
Interdental septum and interradicular septum	3%
Cleft, periodontal situation	4%
Periradicular and periapical bone structure	14%
Image section is complete. Admissible?	20%
Image section compliant to the justifying indication	16%
Quality defects are relevant for the assessment	84%
Secondary/incidental findings	39%

When assessing the radiographs with regard to the justifying indication, the field of view was sufficiently covered in 107 (84%). In 20 (16%), the image section was not completely adequate. Secondary findings were noted from 78 (61%) radiographs. Data on the 20 images (16%) with clear quality deficiencies are presented in Table 3.

**TABLE 3:** Number *N* of quality defects found.

Quality defects	*N*	%
Dental radiograph setting is too steep	1	1%
Overexposed radiograph	11	9%
Superimposition in radiograph	4	3%
Dental phosphor storage plate is not aligned occlusally	54	43%
Quality of the phosphor storage plate	60	47%
Center point of the X‐ray beam is not centred	36	28%
Foreign bodies: superimposition of foreign bodies in the X‐ray path	0	0%
Motion artefacts	5	4%

**FIGURE 1:**
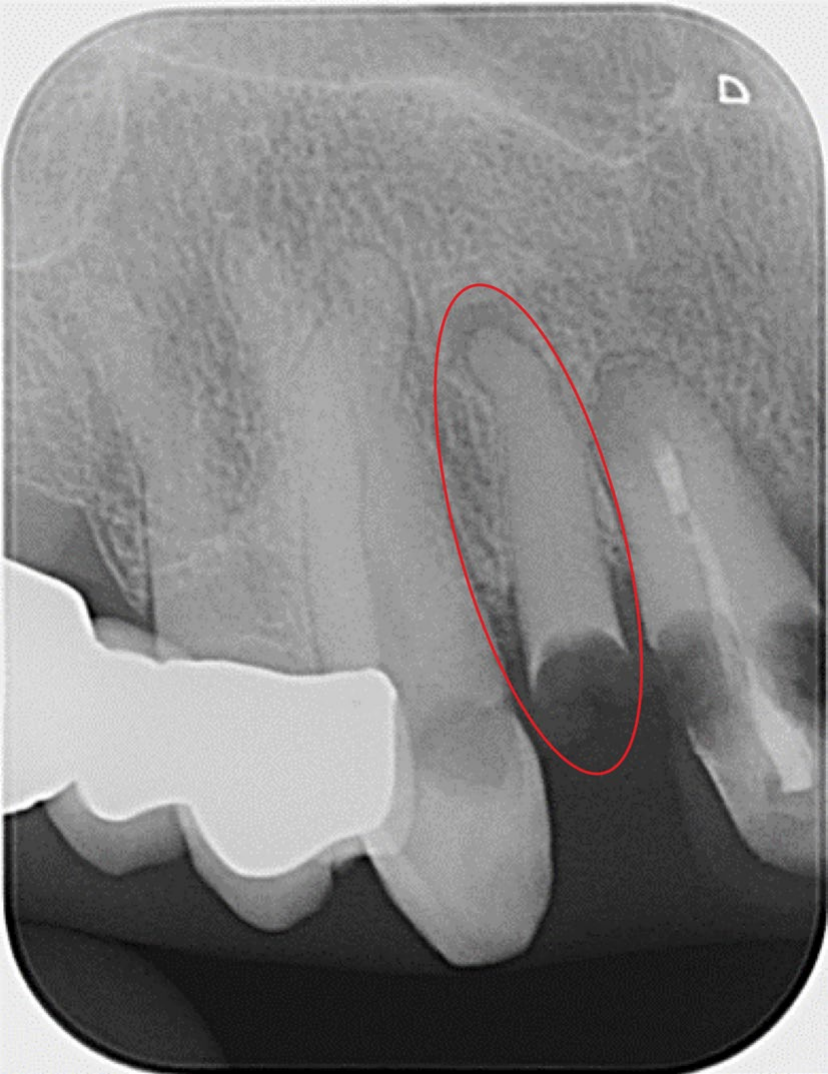
An example of a high‐quality radiograph. In addition, there is a secondary finding (highlighted in red) which, in our opinion, is decisive for the dental diagnosis and treatment. Findings: Tooth 11 with insufficient root canal filling and extensive caries on the mesial and distal aspect of the tooth. Caries on mesial side of tooth 13. Clinically invisible, secondary finding of a root remnant of tooth 12 with apical periodontitis. [Colour figure can be viewed at wileyonlinelibrary.com]

**FIGURE 2:**
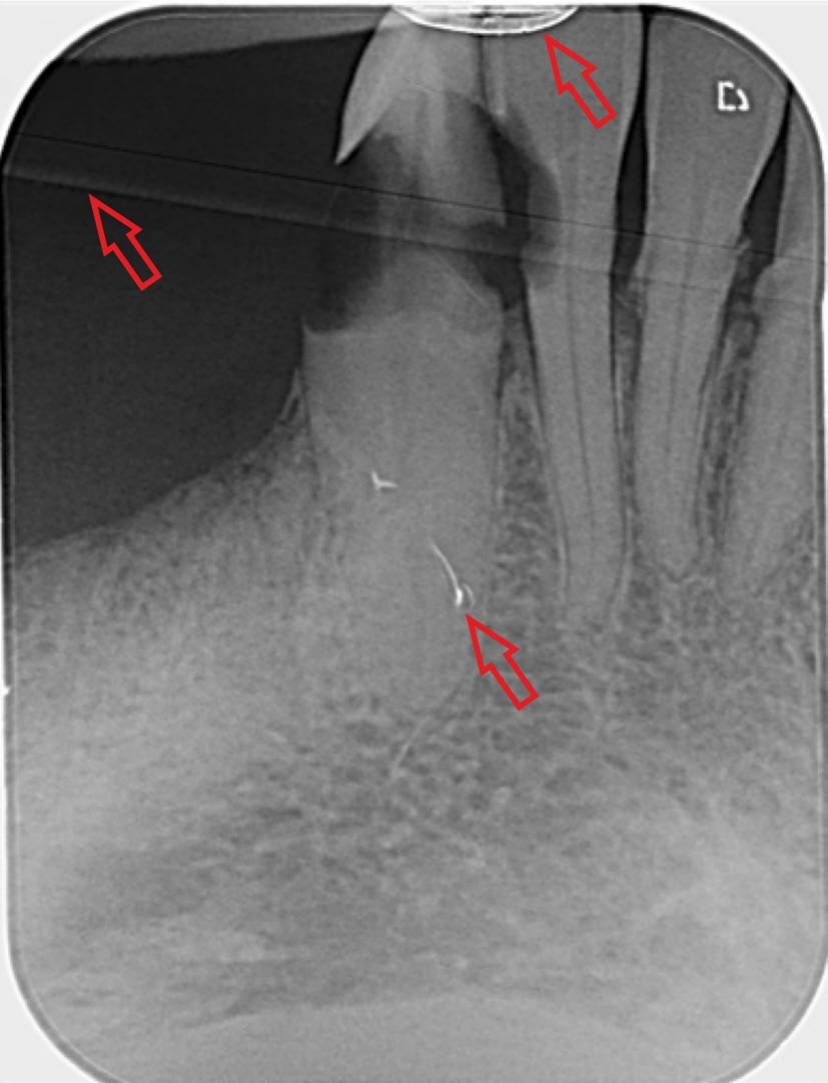
An example of a dental radiograph with quality defects of the image plate or PSP [scratches, folds and wear at the edge (see red arrows)], which are not relevant for the diagnosis [12, 15]. Findings: Teeth 42, 43 with carious destruction of the crown (tooth 43) and extensive caries on the distal aspect of the tooth (tooth 42). Secondary finding: Dental calculus on teeth 41 and 31. Widened periodontal space on tooth 41. [Colour figure can be viewed at wileyonlinelibrary.com]

The dental radiographs (Figures 1, 2) illustrate different image quality issues in mobile dental radiology with the handheld method.

## 4 Discussion

In this study, we investigated 107 of 127 dental radiographs (83%) in the anterior, premolar and molar regions, which showed no diagnostically relevant quality defects, and found out that we achieved the quality standards published by other authors under inpatient conditions [15]. According to the guidelines for dental single tooth radiographs of the German Federal Dental Association, dental radiographs should show the entire tooth to be examined—from the crown to the apex—as well as the periodontal bone structure and the interradicular septum [12, 15, 16]. These quality criteria were met in over 96% of dental radiographs, which is even better than 83% in normal environments [15, 17]. In 84% of dental radiographs, the field of view corresponded to the justifying indication, comparable to 86% in normal environments [15]. Overall, our study demonstrates that a portable dental X‐ray unit achieves comparable results to a wall‐mounted intraoral X‐ray unit. This study's findings are also consistent with investigations by other work groups, which have dealt with the advantages and disadvantages of mobile X‐ray devices [11, 16, 17, 18]. In this study, we detected 16% of qualitative defects under mobile conditions, which is the same range as under stationary conditions (17%) [15]. These quality defects could be further reduced by using direct digital detectors or through strict quality control of the PSPs. The use of a positioning system for image plates should be considered by the user depending on the situation. Motion artefacts were detected in 4% of cases. Given the expected difficulties in taking radiographs in domiciliary dental care, especially with patients with limited compliance, this result can be considered as acceptable. We found no evidence of additional motion artefacts caused by the physician using the handheld unit compared to a wall‐mounted unit. The shorter exposure time of 0.12 s in the anterior region and 0.16 s in the molar region (vs 0.25 and 0.32 s, respectively, compared to a stationary unit) may even contribute to a reduction in motion artefacts.

This study is not a direct comparison between handheld and wall‐mounted X‐ray units. The study of Lommen et al. reported a significantly better perpendicularity of the produced dental radiographs for the handheld Nomad Pro 2 [17]. Other studies examining bitewing radiographs and images of the molar and premolar region for quality differences between the Nomad Pro 2 and the Heliodent Plus wall‐mounted unit showed no significant differences between the X‐ray units [11, 16, 18].

According to Nietschke et al., the operator's training and experience are crucial for image quality [16]. In order to maintain consistent image quality, all 127 dental radiographs in the present study were taken by the same experienced dentist. The influence of professional experience on the ability to make radiological diagnoses has been extensively studied [16]. To minimise this influence, the image quality assessment was performed using a grading system by three dentists with different specialisations and professional experience. As shown in Table 11, appropriate grading systems are often used for the subjective assessment of radiographs [19, 20, 21, 22, 23, 24, 25]. Dental care in nursing homes presents significant challenges, particularly with regard to oral preventive medicine. Surgical procedures, in particular, are legally contestable without dental radiology, and this raises professional and ethical concerns.

In our study, treatment‐relevant (unexpected) secondary findings or incidental findings such as osteolysis or root remnants were observed in 61% of cases. The advantage of mobile radiology is that the treating dentist can make a more precise dental, oral and maxillofacial diagnosis directly at the patient's bedside, even for patients with limited compliance and/or in a demanding environment, such as retirement or nursing homes. To ensure the same quality standards in mobile dentistry as in inpatient care, mobile intraoral radiography is essential. In conclusion, this study demonstrated that portable X‐ray machines can meet the quality requirements for intraoral X‐ray images and are comparable to conventional wall‐mounted dental X‐ray machines in terms of image quality.

## Author Contributions

The authors contributed as follows: Amina Maria Geibel, Constanze Keutel, Margrit‐Ann Geibel conceptualization, investigation; Amina Maria Geibel writing – original draft; Amina Maria Geibel, Constanze Keutel, Daniela Kildal, Margrit‐Ann Geibel data curation, formal analysis, methodology, validation, visualization, writing – review and editing. All authors have reviewed and agreed to the published version of the manuscript.

## Ethics Statement

This study was performed according to the Helsinki Declaration, approved by the Ethics Committee of University Ulm (no. 149/09). All participants were informed, and the treating physician has obtained the person's free, informed consent.

## Conflicts of Interest

There is no Conflicts of Interest with the company Dexis/KaVo. There was no funding in the form of monetary payments for this study.

## Data Availability

The data that support the findings of this study are available on request from the corresponding author. The data are not publicly available due to privacy or ethical restrictions.
